# (4*Z*)-4-[(Cyclo­propyl­amino)(phen­yl)methyl­ene]-3-methyl-1-phenyl-1*H*-pyrazol-5(4*H*)-one

**DOI:** 10.1107/S1600536810013723

**Published:** 2010-04-21

**Authors:** Hai-Zhen Xu, Yan-Xia Yang, You-Quan Zhu

**Affiliations:** a College of Chemistry, Tianjin Normal University, 393 Binshuixi Road, Xiqing District, Tianjin 300387, People’s Republic of China; b Nankai High School, 100 Sima Road, Nankai District, Tianjin 300100, People’s Republic of China; c State Key Laboratory of Elemento-Organic Chemistry, Nankai University, Tianjin 300071, People’s Republic of China

## Abstract

In the title compound, C_20_H_19_N_3_O, the dihedral angles formed by the pyrazolone ring with the two phenyl rings are 64.27 (6) and 17.00 (6)°. The mol­ecular structure is stabilized by intra­molecular N—H⋯O and C—H⋯O hydrogen bonds. In the crystal, the mol­ecules are linked into chains along the *b* axis by inter­molecular C—H⋯O hydrogen bonds.

## Related literature

For the anti­bacterial, biological and analgesic activity of metal complexes of 1-phenyl-3-methyl-4-benzoyl­pyrazolon-5-one, see: Li *et al.* (1997 ▶); Liu *et al.* (1980 ▶); Zhou *et al.* (1999 ▶). For a related structure, see: Wang *et al.* (2003 ▶).
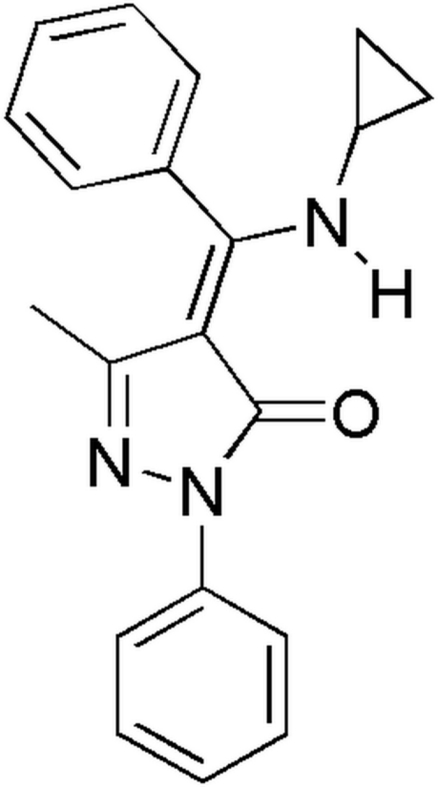


## Experimental

### 

#### Crystal data

C_20_H_19_N_3_O
*M* *_r_* = 317.38
Orthorhombic, 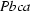

*a* = 8.9790 (18) Å
*b* = 18.500 (4) Å
*c* = 20.050 (4) Å
*V* = 3330.5 (12) Å^3^
*Z* = 8
Cu *K*α radiation
μ = 0.63 mm^−1^
*T* = 113 K
0.24 × 0.21 × 0.20 mm

#### Data collection

Rigaku Saturn70 diffractometer
Absorption correction: multi-scan (*CrystalClear*; Rigaku, 2005 ▶) *T*_min_ = 0.863, *T*_max_ = 0.884
34928 measured reflections
3262 independent reflections
2946 reflections with *I* > 2σ(*I*)
*R*_int_ = 0.060

#### Refinement

*R*[*F* ^2^ > 2σ(*F* ^2^)] = 0.040
*wR*(*F* ^2^) = 0.104
*S* = 1.06
3262 reflections
222 parameters
H atoms treated by a mixture of independent and constrained refinement
Δρ_max_ = 0.18 e Å^−3^
Δρ_min_ = −0.24 e Å^−3^

### 

Data collection: *CrystalClear* (Rigaku, 2005 ▶); cell refinement: *CrystalClear*; data reduction: *CrystalClear*; program(s) used to solve structure: *SHELXS97* (Sheldrick, 2008 ▶); program(s) used to refine structure: *SHELXL97* (Sheldrick, 2008 ▶); molecular graphics: *CrystalStructure* (Rigaku, 2005 ▶); software used to prepare material for publication: *CrystalStructure*.

## Supplementary Material

Crystal structure: contains datablocks global, I. DOI: 10.1107/S1600536810013723/ci5077sup1.cif

Structure factors: contains datablocks I. DOI: 10.1107/S1600536810013723/ci5077Isup2.hkl

Additional supplementary materials:  crystallographic information; 3D view; checkCIF report

## Figures and Tables

**Table 1:** Hydrogen-bond geometry (Å, °)

*D*—H⋯*A*	*D*—H	H⋯*A*	*D*⋯*A*	*D*—H⋯*A*
N3—H1⋯O1	0.92 (2)	1.88 (2)	2.6726 (15)	143 (2)
C20—H20⋯O1	0.95	2.36	2.9453 (16)	120
C10—H10⋯O1^i^	0.95	2.30	3.1809 (17)	154

Symmetry code: (i) 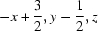.

**Fig. 1.**
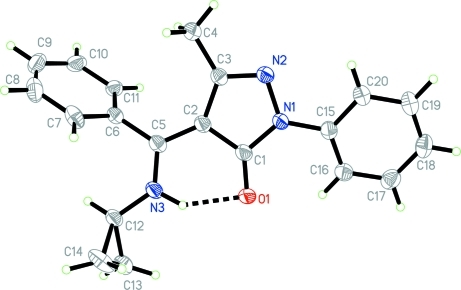
Molecular structure of the title compound, with displacement ellipsoids drawn at the 50% probability level. The dashed line indicates a hydrogen bond.

**Fig. 2.**
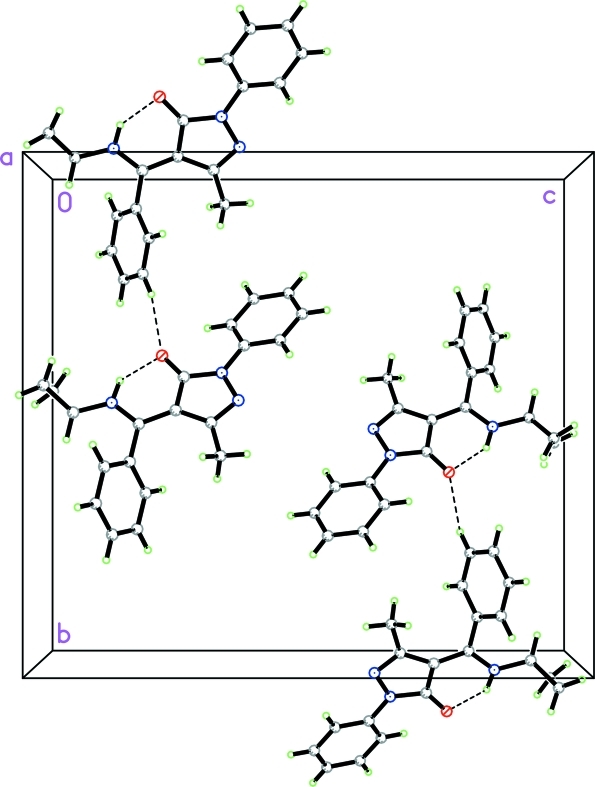
Part of hydrogen-bonded (dashed line) chains in the title compound.

## supplementary crystallographic information

### Comment

1-Phenyl-3-methyl-4-benzoylpyrazolon-5-one (HPMBP), an effective β-diketonate, is widely used and well known for its extractive ability. In recent years, HPMBP and its metal complexes have also been found to have good antibacterial and biological properties. Its metal complexes have analgesic activity (Liu *et al.*, 1980; Li *et al.*, 1997; Zhou *et al.*, 1999). In order to develop new medicines, we have synthesized the title compound and its crystal structure is reported here.

The structure of the title molecule is shown in Fig. 1. The dihedral angles formed by the pyrazolone ring with the C6–C11 and C15–C20 phenyl rings and cyclopropane ring are 64.27 (6)°, 17.00 (6)° and 71.28 (11)°, respectively. The O atom of the 3-methyl-1-phenylpyrazol-5-one moiety and the N atom of the amino group are available for coordination with metals. Atoms O1, C1, C2, C5 and N3 are coplanar (r.m.s. deviation = 0.028 Å). The dihedral angle between this plane and the pyrazoline ring is 4.34 (7)°, close to the value of 3.56 (3)° found in 4-{[3,4-dihydro-5-methyl-3-oxo-2-phenyl-2*H*-pyrazol-4-ylidene(phenyl) methylamino}-1,5-dimethyl-2-phenyl-1*H*-pyrazol-3(2*H*)-one (Wang *et al.*, 2003). The bond lengths within this part of the molecule lie between classical single- and double-bond lengths, indicating extensive conjugation. A strong intramolecular N3—H1···O1 hydrogen bond (Table 1) is observed, leading to a keto-enamine form. The molecule is further stabilized by a C—H···O weak intramolecular hydrogen bond (Table 1).

The crystal structure also involves weak intermolecular C—H···O hydrogen-bond interactions (Fig. 2).

### Experimental

The title compound was synthesized by refluxing a mixture of 1-phenyl-3- methyl-4-benzoylpyrazol-5-one (10 mmol) and cyclopropanamine (10 mmol) in ethanol (80 ml) over a steam bath for about 16 h. Excess solvent was removed by evaporation and the solution was cooled to room temperature. After 2 d, a colourless solid was obtained and this was dried in air. The product was recrystallized from ethanol, to afford colourless crystals of the title compound suitable for X-ray analysis.

### Refinement

C-bonded H atoms were positioned geometrically, with C–H = 0.95–1.00 Å and the amine H atom (H1) was found in a difference map. The amine H atom was refined freely, while C-bonded H atoms were included in the final cycles of refinement using a riding model, with *U*_iso_(H) = 1.2*U*_eq_(CH_2_ and CH) or 1.5*U*_eq_(CH_3_).

### Crystal data

**Table:**

C_20_H_19_N_3_O	*F*(000) = 1344
*M**_r_* = 317.38	*D*_x_ = 1.266 Mg m^−^^3^
Orthorhombic, *P**b**c**a*	Cu *K*α radiation, λ = 1.54187 Å
Hall symbol: -P 2ac 2ab	Cell parameters from 2126 reflections
*a* = 8.9790 (18) Å	θ = 2.2–45.5°
*b* = 18.500 (4) Å	µ = 0.63 mm^−^^1^
*c* = 20.050 (4) Å	*T* = 113 K
*V* = 3330.5 (12) Å^3^	Prism, colourless
*Z* = 8	0.24 × 0.21 × 0.20 mm

### Data collection

**Table:**

Rigaku Saturn70 diffractometer	3262 independent reflections
Radiation source: fine-focus sealed tube	2946 reflections with *I* > 2σ(*I*)
graphite	*R*_int_ = 0.060
ω scans	θ_max_ = 72.5°, θ_min_ = 4.4°
Absorption correction: multi-scan (*CrystalClear*; Rigaku, 2005)	*h* = −8→10
*T*_min_ = 0.863, *T*_max_ = 0.884	*k* = −22→22
34928 measured reflections	*l* = −24→24

### Refinement

**Table:**

Refinement on *F*^2^	Primary atom site location: structure-invariant direct methods
Least-squares matrix: full	Secondary atom site location: difference Fourier map
*R*[*F*^2^ > 2σ(*F*^2^)] = 0.040	Hydrogen site location: inferred from neighbouring sites
*wR*(*F*^2^) = 0.104	H atoms treated by a mixture of independent and constrained refinement
*S* = 1.06	*w* = 1/[σ^2^(*F*_o_^2^) + (0.0583*P*)^2^ + 0.7718*P*] where *P* = (*F*_o_^2^ + 2*F*_c_^2^)/3
3262 reflections	(Δ/σ)_max_ = 0.001
222 parameters	Δρ_max_ = 0.18 e Å^−^^3^
0 restraints	Δρ_min_ = −0.24 e Å^−^^3^

### Special details

**Table:**

Geometry. All esds (except the esd in the dihedral angle between two l.s. planes) are estimated using the full covariance matrix. The cell esds are taken into account individually in the estimation of esds in distances, angles and torsion angles; correlations between esds in cell parameters are only used when they are defined by crystal symmetry. An approximate (isotropic) treatment of cell esds is used for estimating esds involving l.s. planes.
Refinement. Refinement of F^2^ against ALL reflections. The weighted R-factor wR and goodness of fit S are based on F^2^, conventional R-factors R are based on F, with F set to zero for negative F^2^. The threshold expression of F^2^ > 2sigma(F^2^) is used only for calculating R-factors(gt) etc. and is not relevant to the choice of reflections for refinement. R-factors based on F^2^ are statistically about twice as large as those based on F, and R- factors based on ALL data will be even larger.

### Fractional atomic coordinates and isotropic or equivalent isotropic displacement parameters (Å^2^)

**Table:**

	*x*	*y*	*z*	*U*_iso_*/*U*_eq_	
O1	0.67690 (13)	0.11712 (5)	0.26555 (4)	0.0415 (3)	
N1	0.73850 (11)	0.08273 (5)	0.15606 (5)	0.0274 (2)	
N2	0.82349 (11)	0.02871 (5)	0.12501 (5)	0.0262 (2)	
N3	0.79592 (14)	0.02403 (5)	0.35244 (5)	0.0336 (3)	
C1	0.73711 (15)	0.07497 (6)	0.22477 (6)	0.0299 (3)	
C2	0.82015 (14)	0.00961 (6)	0.23704 (5)	0.0266 (3)	
C3	0.87030 (13)	−0.01421 (6)	0.17276 (5)	0.0248 (2)	
C4	0.96427 (14)	−0.07788 (7)	0.15470 (6)	0.0313 (3)	
H4A	0.9070	−0.1224	0.1613	0.038*	
H4B	1.0531	−0.0789	0.1831	0.038*	
H4C	0.9944	−0.0742	0.1079	0.038*	
C5	0.84118 (13)	−0.01694 (6)	0.30210 (5)	0.0260 (3)	
C6	0.90512 (12)	−0.08952 (6)	0.31601 (5)	0.0246 (2)	
C7	1.03261 (14)	−0.09759 (8)	0.35553 (6)	0.0344 (3)	
H7	1.0806	−0.0562	0.3736	0.041*	
C8	1.08875 (16)	−0.16611 (9)	0.36826 (7)	0.0454 (4)	
H8	1.1754	−0.1715	0.3950	0.054*	
C9	1.01929 (17)	−0.22661 (8)	0.34232 (7)	0.0465 (4)	
H9	1.0586	−0.2733	0.3512	0.056*	
C10	0.89239 (16)	−0.21934 (7)	0.30344 (6)	0.0379 (3)	
H10	0.8443	−0.2610	0.2860	0.045*	
C11	0.83581 (13)	−0.15080 (6)	0.29005 (6)	0.0269 (3)	
H11	0.7495	−0.1458	0.2631	0.032*	
C12	0.78672 (15)	0.00510 (6)	0.42190 (6)	0.0319 (3)	
H12	0.7987	−0.0472	0.4331	0.038*	
C13	0.84272 (17)	0.05784 (9)	0.47193 (6)	0.0446 (4)	
H13A	0.8899	0.0384	0.5128	0.053*	
H13B	0.8863	0.1035	0.4552	0.053*	
C14	0.68020 (16)	0.04703 (8)	0.46359 (6)	0.0378 (3)	
H14A	0.6226	0.0860	0.4417	0.045*	
H14B	0.6263	0.0209	0.4993	0.045*	
C15	0.67274 (13)	0.13754 (6)	0.11655 (6)	0.0269 (3)	
C16	0.72259 (13)	0.14729 (6)	0.05108 (6)	0.0290 (3)	
H16	0.8004	0.1180	0.0338	0.035*	
C17	0.65736 (14)	0.20020 (7)	0.01143 (6)	0.0346 (3)	
H17	0.6903	0.2064	−0.0332	0.042*	
C18	0.54501 (15)	0.24393 (7)	0.03623 (7)	0.0369 (3)	
H18	0.5017	0.2803	0.0090	0.044*	
C19	0.49628 (15)	0.23398 (7)	0.10137 (7)	0.0364 (3)	
H19	0.4194	0.2639	0.1185	0.044*	
C20	0.55835 (14)	0.18084 (6)	0.14184 (6)	0.0322 (3)	
H20	0.5234	0.1741	0.1861	0.039*	
H1	0.7554 (18)	0.0673 (10)	0.3395 (8)	0.048 (4)*	

### Atomic displacement parameters (Å^2^)

**Table:**

	*U*^11^	*U*^22^	*U*^33^	*U*^12^	*U*^13^	*U*^23^
O1	0.0784 (7)	0.0231 (4)	0.0230 (4)	0.0114 (4)	0.0030 (4)	−0.0021 (3)
N1	0.0387 (6)	0.0230 (5)	0.0204 (5)	0.0014 (4)	−0.0011 (4)	−0.0008 (3)
N2	0.0302 (5)	0.0259 (5)	0.0226 (5)	−0.0004 (4)	−0.0001 (4)	−0.0016 (4)
N3	0.0610 (7)	0.0201 (5)	0.0197 (5)	−0.0004 (4)	−0.0005 (4)	0.0003 (4)
C1	0.0472 (7)	0.0215 (5)	0.0210 (5)	−0.0018 (5)	−0.0007 (5)	−0.0012 (4)
C2	0.0376 (6)	0.0207 (5)	0.0216 (6)	−0.0036 (4)	−0.0013 (4)	−0.0009 (4)
C3	0.0266 (6)	0.0253 (5)	0.0225 (5)	−0.0039 (4)	−0.0013 (4)	−0.0006 (4)
C4	0.0315 (6)	0.0358 (6)	0.0267 (6)	0.0052 (5)	0.0025 (5)	0.0019 (5)
C5	0.0341 (6)	0.0221 (5)	0.0217 (5)	−0.0075 (4)	−0.0014 (4)	−0.0011 (4)
C6	0.0268 (6)	0.0264 (5)	0.0204 (5)	−0.0019 (4)	0.0012 (4)	0.0017 (4)
C7	0.0273 (6)	0.0524 (8)	0.0235 (5)	−0.0080 (5)	−0.0008 (5)	0.0058 (5)
C8	0.0305 (7)	0.0732 (10)	0.0325 (7)	0.0154 (6)	0.0044 (5)	0.0204 (7)
C9	0.0528 (9)	0.0464 (8)	0.0403 (7)	0.0257 (7)	0.0173 (6)	0.0171 (6)
C10	0.0528 (8)	0.0255 (6)	0.0353 (6)	0.0064 (5)	0.0129 (6)	0.0016 (5)
C11	0.0297 (6)	0.0241 (5)	0.0269 (6)	−0.0004 (4)	0.0018 (4)	−0.0016 (4)
C12	0.0509 (8)	0.0256 (6)	0.0191 (5)	−0.0011 (5)	0.0004 (5)	0.0013 (4)
C13	0.0500 (8)	0.0613 (9)	0.0223 (6)	−0.0220 (7)	0.0007 (5)	−0.0058 (6)
C14	0.0435 (8)	0.0431 (7)	0.0268 (6)	0.0033 (6)	0.0029 (5)	0.0014 (5)
C15	0.0332 (6)	0.0225 (5)	0.0249 (5)	−0.0050 (4)	−0.0058 (4)	−0.0005 (4)
C16	0.0289 (6)	0.0309 (6)	0.0273 (6)	−0.0038 (4)	−0.0025 (4)	0.0033 (5)
C17	0.0336 (7)	0.0391 (7)	0.0310 (6)	−0.0064 (5)	−0.0050 (5)	0.0102 (5)
C18	0.0370 (7)	0.0328 (6)	0.0410 (7)	−0.0004 (5)	−0.0110 (5)	0.0084 (5)
C19	0.0372 (7)	0.0323 (6)	0.0397 (7)	0.0035 (5)	−0.0076 (5)	−0.0035 (5)
C20	0.0394 (7)	0.0293 (6)	0.0280 (6)	−0.0006 (5)	−0.0035 (5)	−0.0033 (5)

### Geometric parameters (Å, °)

**Table:**

O1—C1	1.2525 (15)	C9—H9	0.95
N1—C1	1.3852 (15)	C10—C11	1.3921 (17)
N1—N2	1.4029 (13)	C10—H10	0.95
N1—C15	1.4158 (14)	C11—H11	0.95
N2—C3	1.3130 (15)	C12—C13	1.4869 (17)
N3—C5	1.3260 (15)	C12—C14	1.4885 (18)
N3—C12	1.4382 (15)	C12—H12	1.00
N3—H1	0.917 (18)	C13—C14	1.482 (2)
C1—C2	1.4417 (16)	C13—H13A	0.99
C2—C5	1.4066 (15)	C13—H13B	0.99
C2—C3	1.4345 (15)	C14—H14A	0.99
C3—C4	1.4935 (16)	C14—H14B	0.99
C4—H4A	0.98	C15—C20	1.3978 (17)
C4—H4B	0.98	C15—C16	1.3984 (17)
C4—H4C	0.98	C16—C17	1.3904 (17)
C5—C6	1.4868 (16)	C16—H16	0.95
C6—C11	1.3940 (16)	C17—C18	1.3854 (19)
C6—C7	1.4001 (16)	C17—H17	0.95
C7—C8	1.388 (2)	C18—C19	1.3898 (19)
C7—H7	0.95	C18—H18	0.95
C8—C9	1.383 (2)	C19—C20	1.3912 (17)
C8—H8	0.95	C19—H19	0.95
C9—C10	1.387 (2)	C20—H20	0.95
			
C1—N1—N2	111.86 (9)	C10—C11—C6	120.38 (12)
C1—N1—C15	128.83 (10)	C10—C11—H11	119.8
N2—N1—C15	119.26 (9)	C6—C11—H11	119.8
C3—N2—N1	106.35 (9)	N3—C12—C13	118.30 (11)
C5—N3—C12	127.99 (10)	N3—C12—C14	116.99 (11)
C5—N3—H1	113.9 (10)	C13—C12—C14	59.76 (9)
C12—N3—H1	117.6 (10)	N3—C12—H12	116.6
O1—C1—N1	126.06 (11)	C13—C12—H12	116.6
O1—C1—C2	129.34 (11)	C14—C12—H12	116.6
N1—C1—C2	104.59 (10)	C14—C13—C12	60.17 (9)
C5—C2—C3	133.14 (11)	C14—C13—H13A	117.8
C5—C2—C1	121.37 (10)	C12—C13—H13A	117.8
C3—C2—C1	105.47 (9)	C14—C13—H13B	117.8
N2—C3—C2	111.64 (10)	C12—C13—H13B	117.8
N2—C3—C4	118.76 (10)	H13A—C13—H13B	114.9
C2—C3—C4	129.60 (10)	C13—C14—C12	60.07 (9)
C3—C4—H4A	109.5	C13—C14—H14A	117.8
C3—C4—H4B	109.5	C12—C14—H14A	117.8
H4A—C4—H4B	109.5	C13—C14—H14B	117.8
C3—C4—H4C	109.5	C12—C14—H14B	117.8
H4A—C4—H4C	109.5	H14A—C14—H14B	114.9
H4B—C4—H4C	109.5	C20—C15—C16	120.12 (11)
N3—C5—C2	117.73 (11)	C20—C15—N1	120.92 (10)
N3—C5—C6	119.45 (10)	C16—C15—N1	118.95 (11)
C2—C5—C6	122.76 (10)	C17—C16—C15	119.52 (12)
C11—C6—C7	119.31 (11)	C17—C16—H16	120.2
C11—C6—C5	119.47 (10)	C15—C16—H16	120.2
C7—C6—C5	121.21 (10)	C18—C17—C16	120.84 (12)
C8—C7—C6	119.90 (12)	C18—C17—H17	119.6
C8—C7—H7	120.0	C16—C17—H17	119.6
C6—C7—H7	120.0	C17—C18—C19	119.29 (11)
C9—C8—C7	120.41 (13)	C17—C18—H18	120.4
C9—C8—H8	119.8	C19—C18—H18	120.4
C7—C8—H8	119.8	C18—C19—C20	121.04 (12)
C8—C9—C10	120.22 (12)	C18—C19—H19	119.5
C8—C9—H9	119.9	C20—C19—H19	119.5
C10—C9—H9	119.9	C19—C20—C15	119.19 (12)
C9—C10—C11	119.77 (13)	C19—C20—H20	120.4
C9—C10—H10	120.1	C15—C20—H20	120.4
C11—C10—H10	120.1		
			
C1—N1—N2—C3	2.41 (13)	C2—C5—C6—C7	122.97 (13)
C15—N1—N2—C3	179.94 (10)	C11—C6—C7—C8	0.19 (17)
N2—N1—C1—O1	176.20 (12)	C5—C6—C7—C8	179.28 (11)
C15—N1—C1—O1	−1.0 (2)	C6—C7—C8—C9	−0.20 (19)
N2—N1—C1—C2	−3.16 (13)	C7—C8—C9—C10	−0.2 (2)
C15—N1—C1—C2	179.61 (11)	C8—C9—C10—C11	0.60 (19)
O1—C1—C2—C5	1.6 (2)	C9—C10—C11—C6	−0.61 (18)
N1—C1—C2—C5	−179.04 (10)	C7—C6—C11—C10	0.21 (17)
O1—C1—C2—C3	−176.70 (13)	C5—C6—C11—C10	−178.89 (11)
N1—C1—C2—C3	2.63 (13)	C5—N3—C12—C13	135.28 (14)
N1—N2—C3—C2	−0.57 (13)	C5—N3—C12—C14	−156.29 (13)
N1—N2—C3—C4	179.70 (10)	N3—C12—C13—C14	106.42 (13)
C5—C2—C3—N2	−179.36 (12)	N3—C12—C14—C13	−108.60 (13)
C1—C2—C3—N2	−1.32 (13)	C1—N1—C15—C20	−19.43 (18)
C5—C2—C3—C4	0.3 (2)	N2—N1—C15—C20	163.52 (10)
C1—C2—C3—C4	178.36 (11)	C1—N1—C15—C16	161.44 (12)
C12—N3—C5—C2	170.89 (12)	N2—N1—C15—C16	−15.61 (15)
C12—N3—C5—C6	−6.43 (19)	C20—C15—C16—C17	−0.12 (17)
C3—C2—C5—N3	170.75 (13)	N1—C15—C16—C17	179.02 (10)
C1—C2—C5—N3	−7.04 (17)	C15—C16—C17—C18	0.82 (18)
C3—C2—C5—C6	−12.0 (2)	C16—C17—C18—C19	−0.70 (19)
C1—C2—C5—C6	170.18 (11)	C17—C18—C19—C20	−0.11 (19)
N3—C5—C6—C11	119.23 (13)	C18—C19—C20—C15	0.80 (19)
C2—C5—C6—C11	−57.95 (16)	C16—C15—C20—C19	−0.68 (17)
N3—C5—C6—C7	−59.85 (16)	N1—C15—C20—C19	−179.80 (11)

### Hydrogen-bond geometry (Å, °)

**Table:**

*D*—H···*A*	*D*—H	H···*A*	*D*···*A*	*D*—H···*A*
N3—H1···O1	0.92 (2)	1.88 (2)	2.6726 (15)	143 (2)
C20—H20···O1	0.95	2.36	2.9453 (16)	120
C10—H10···O1^i^	0.95	2.30	3.1809 (17)	154

Symmetry codes: (i) −*x*+3/2, *y*−1/2, *z*.

## References

[bb1] Li, J.-Z., Yu, W.-J. & Du, X.-Y. (1997). *Chin. J. Appl. Chem.* **14**, 98–100.

[bb2] Liu, J.-M., Yang, R.-D. & Ma, T.-R. (1980). *Chem. J. Chin. Univ.* **1**, 23–29.

[bb3] Rigaku (2005). *CrystalClear* and *CrystalStructure* Rigaku Corporation, Tokyo, Japan.

[bb4] Sheldrick, G. M. (2008). *Acta Cryst.* A**64**, 112–122. 10.1107/S0108767307043930 18156677

[bb5] Wang, J.-L., Yang, Y., Zhang, X. & Miao, F.-M. (2003). *Acta Cryst.* E**59**, o430–o432.

[bb6] Zhou, Y.-P., Yang, Zh.-Y., Yu, H.-J. & Yang, R.-D. (1999). *Chin. J. Appl. Chem.* **16**, 37–41.

